# Automated Structural Bolt Micro Looseness Monitoring Method Using Deep Learning

**DOI:** 10.3390/s24227340

**Published:** 2024-11-18

**Authors:** Min Qin, Zhenbo Xie, Jing Xie, Xiaolin Yu, Zhongyuan Ma, Jinrui Wang

**Affiliations:** 1 Qingdao Campus of Naval Aviation University, Qingdao 266041, China; 2 National University of Defense Technology, Changsha 410073, China; 3 College of Mechanical and Electronic Engineering, Shandong University of Science and Technology, Qingdao 266590, China

**Keywords:** bolt micro looseness monitoring, characterization function, stacked auto-encoders, batch normalization

## Abstract

The detection of bolt loosening in key components of aircraft engines faces problems such as complex and difficult-to-establish bolt loosening mechanism models, difficulty in identifying early loosening, and difficulty in extracting signal features with nonlinear and non-stationary characteristics. Therefore, the automated structural bolt micro looseness monitoring method using deep learning was proposed. Specifically, the addition of batch normalization methods enables the established Batch Normalized Stacked Autoencoders (BNSAEs) model to converge quickly and effectively, making the model easy to build and effective. Additionally, using characterization functions preprocess the original response signal not only simplifies the data structure but also ensures the integrity of features, which is beneficial for network training and reduces time costs. Finally, the effectiveness of the proposed method was verified by taking the bolted connection structures of two key components of aircraft engines, namely bolt connection structures and flange connection structures, as examples.

## 1. Introduction

**Figure 1:**
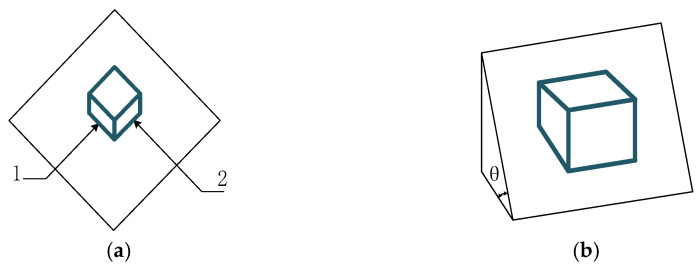
Explanation model for bolt loosening mechanism. (**a**) plane model (**b**) slope model.

**Figure 2:**
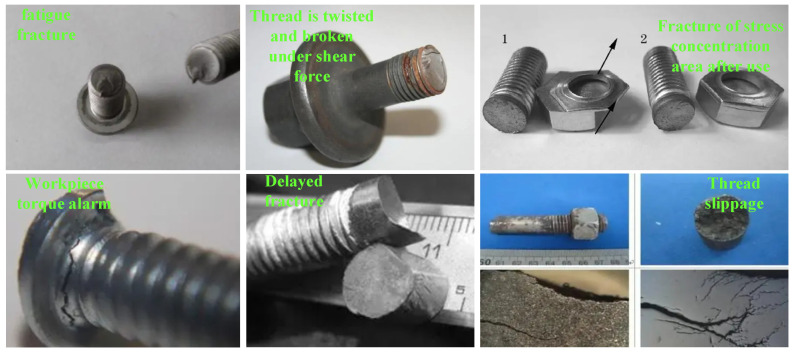
Bolt failure mode.

Owing to numerous strengths, such as convenient construction and low cost, bolted connections have been widely employed in the civil, mechanical, chemical, and aerospace industries. However, the bolt connection structure bears the vibration, impact, and fatigue caused by the alternating loads for a long time, resulting in the loosening or even falling off of the bolt, whose failure may cause disastrous consequences [1,2]. The mechanism of bolt loosening is shown in Figure 1. As shown in Figure 1a, when a block is on a plane, if it needs to be moved, a force that can overcome the maximum frictional force can be applied in direction 1. However, if there is such a force in direction 2, only a small force needs to be provided in direction 1 to push the block. As shown in Figure 1b, when the object block is on a slope, due to the inclination angle being smaller than the friction angle, the object block has a self-locking effect and will not slide downwards. But if a lateral excitation is applied to the block, it will slide downwards under the action of gravity. The loosening of bolts is similar to this process, except that gravity is similar to some axial forces, such as bolt pre-tightening force and axial load, and lateral excitation is similar to the lateral load of bolts. The bolt failure mode is shown in Figure 2, which is influenced by multiple factors. Bolt loosening and fatigue fracture are the two most common forms of failure. The strength of bolt connections directly determines the reliability of mechanical structures, so many scholars have focused on the study of bolt failure and achieved commendable results. However, there are still many unresolved issues regarding bolt failure, such as micro looseness.

Aircraft engines are known as the “heart” of airplanes, and their importance is self-evident. If the failure of the bolt detection of key components of aircraft engines is not detected in a timely manner, it can cause engine damage or even flight accidents, which will bring huge economic losses and casualties to the country and the people. For example, high-speed aircraft engines provide power for aircraft flight, but when a bolt on the high-speed rotating turbine disk falls off, the detached bolt may move with inertia, damaging the blades and casing at all levels until it flies out of the aircraft. Therefore, the research on fast and effective bolt-loosening detection methods has important research value for the flight safety of aircraft engines and the protection of national property security. Therefore, establishing a reliable and effective monitoring method is important for the research value of flight safety of aircraft engines and the protection of national property security.

With the rapid growth of structural health monitoring (SHM) methods [3,4,5], researchers have developed several methods to identify bolt looseness. For instance, the ultrasonic-based method, which has received much concern recently, is still hindered in its practical application by virtue of its dependence on issues with sensor deployment [6]. For this reason, the vision-based method emerges as time requires, and it only needs a simple and low-cost setup and operation [7]. However, the undifferentiated change of bolt aspect during the early looseness condition of bolted connection often impedes the employ of vision-based techniques. The motion of vision equipment and illumination conditions will also lead to the decline of captured image quality, which will affect the accuracy of the vision-based method. Additionally, researchers found that the acoustic signal generated by tapping the structure corresponds to the variety of mechanical properties caused by structural damage [8], but how to extract the acoustic signal accurately and effectively is a challenge. Vibration signal analysis technology has developed from the primary time-domain and frequency-domain to intelligent data processing methods with learning ability, such as data processing method based on spectral analysis, signal time-frequency domain analysis method, feature extraction method using principal component analysis, and vibration signal processing technology using fractal theory [9]. Just as the development of vibration signal analysis technology, the vibration-based method has been well applied. Nonetheless, the model between bolt axial preload and vibration parameters is complex, and the layout of measuring points also directly affects the detection results, which increases the calculation and labor cost and limits the development of traditional vibration-based methods [10]. The key to bolt micro looseness monitoring method using vibration analysis lies in vibration signal processing technology and feature extraction technology.

Notably, deep learning with strong feature extraction ability has been widely used in adaptive signal processing, language recognition, image recognition, signal detection, fault diagnosis, and pattern recognition [4,11]. As is well known, convolutional neural network (CNN) has been successfully applied in the field of fault diagnosis [12,13]. And apparently, it is advisable to use deep learning instead of traditional complex models. Fault diagnosis and bolt loosening both belong to pattern recognition. Therefore, based on the successful application of deep learning in fault diagnosis, researchers have studied the bolt looseness monitoring method using deep learning. Such as a bolt looseness monitoring method using convolutional long short-term memory networks was proposed by Wang [14], which is the percussion-based method. Ref. [6] proposed a method to monitor bolt looseness with an audio signal using a convolutional neural network (CNN), and the audio signals are collected by the percussion-based method. In addition, the vision-based method based on deep learning has also begun to develop. Ref. [7] proposed a bolt looseness monitoring method using a region-based CNN, and the recognition accuracy is 99%. A network-based Single Shot MultiBox Detector algorithm is established to detect bolt looseness, which is proposed in the vision-based method [15]. Another, Huynh et al. [16] gave a bolt looseness monitoring method based on CNN. Others, Ramana et al. [17], proposed an automated bolt looseness detection method using support vector machines. Moreover, Zhao et al. [18] identified the rotational features of a single bolt appearance using a Single Shot Multibox Detector. However, it has poor practicability in monitoring multiple bolted connections. To solve the above problems, Wang et al. [19] designed another bolt looseness monitoring method using Hough transform line detection. Recently, the achievement of deep learning in numerous fields has opened a new example for SHM [20]. As described above, the robustness and practicality of bolt looseness monitoring have also been strengthened by deep learning. In industrial applications, the lightweight of the model is very important, as Wang et al. [21,22] conducted in-depth research on the application of lightweight models in fault diagnosis.

As is well known, the advantages of SAE are as follows: (1) It can effectively learn multi-level abstract feature representations from raw data. (2) Unsupervised methods can be used to pre-train the model, reducing the data annotation burden of supervised learning tasks. (3) Suitable for dimensionality reduction and feature extraction of large-scale high-dimensional data. Various deep-learning-based algorithms, including the stacked auto-encoder (SAE) and its variants [23,24,25,26], have achieved certain results in the field of SHM. Wei et al. applied the improved stacked autoencoder to the damage detection of the pipeline rack platform [27]. Shao et al. proposed a new SHM for stacked transmission autoencoders based on PSO optimization [28]. Luo et al. proposed the application of transfer learning based on an improved stacked autoencoder in bearing fault diagnosis [29]. However, the drawbacks of SAE are also evident; for example, (1) the training process is complex and requires separate training for each layer of the autoencoder. (2) For complex data distributions and noisy data, it may be difficult to learn useful feature representations. (3) Refactoring erroneous optimization objectives may not always align with downstream task objectives and require further fine-tuning. Fortunately, BN can avoid some of the drawbacks of SAE. BN can reparametrize almost any network elegantly and, to some extent, overcome the drawbacks of SAE [30]. For this purpose, a vibration-based bolt micro looseness monitoring method using BNSAEs is presented, which has strong feature extraction and monitoring ability. Compared with the traditional method, this method directly models the bolt micro looseness and response signals based on unsupervised learning, avoiding the secondary error. Compared with the existing deep learning, the feature function proposed by this method preprocesses the signal, which can preliminarily extract the features and reduce the operation and time cost of the network. The remainder of this literature is structured as follows. The background is detailed in Section 2. The proposed method includes a characterization function, and the BNSAE framework is provided in Section 3. Experimental verification is described in Section 4. Finally, conclusions are given in Section 5.

## 2. Theoretical Background

### 2.1. Auto-Encoder

**Figure 3:**
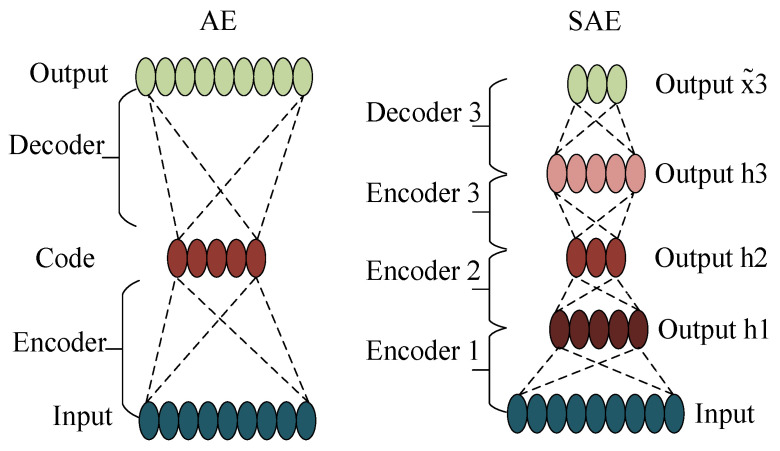
Structure of AE and SAE.

Auto-encoder (AE) includes an encoder and decoder, which was formerly known as auto-association, and constructs the “building block” of deep learning [24]. In other words, it is a system trying to restore its original input, as is shown in Figure 3. The implicit features are used to reconstruct the original input data.

Firstly, given the input of AE, and then the code ***h*** is calculated from **x** using the encoder. The encoder process is as follows,

(1) $$x=(x_{1},x_{2},\cdots ,x_{i},\cdots ,x_{n}),$$

(2) $$h=e(W_{e}x+b_{e}),$$

where *e*(▪) is the encoding function, **W***_e_* and b*_e_* are the weight matrix and bias vector of the encoder, respectively.

Secondly, ***h*** is the input of the decoder, $\tilde{x}$ is calculated from ***h*** using the decoder. The decoder process is as follows,

(3) $$\tilde{x}=d(W_{d}h+b_{d}),$$

(4) $$\tilde{x}=({\tilde{x}}_{1},{\tilde{x}}_{2},\cdots ,{\tilde{x}}_{i},\cdots ,{\tilde{x}}_{n}),$$

where *d*(▪) is the decoding function, **W***_d_* and b*_d_* are the weight matrix and bias vector of the decoder, respectively.

In particular, in the process of encoding and decoding, to make $\tilde{x}$ ≈ **x**, AE needs to adjust the encoding and decoding parameters (**W** and b) according to the objective function. The objective function is as follows,

(5) $$J(W,b)=\frac{1}{n}\sum_{i=1}^{n}{\left(\tilde{x}-x\right)}^{2}.$$

Stacking the AE layer by layer to form a stacked auto-encoder (SAE) [25,26], the specific process is as follows: firstly, given the original input data, the AE’s first layer is trained in an unsupervised manner to reduce the reconstruction error until it reaches the set value. Secondly, the output of the hidden layer of the first AE is taken as the input of the second AE and trained for the second AE in the same way as above. Repeat the above process until all AE are completed, as shown in Figure 3. However, it is an undeniable fact that the training process of SAE is complex and difficult to learn from complex data distributions and noisy data features. Accordingly, avoiding the disadvantages of SAE and leveraging its advantages are the next tasks to be completed.

### 2.2. Batch Normalization

**Figure 4:**
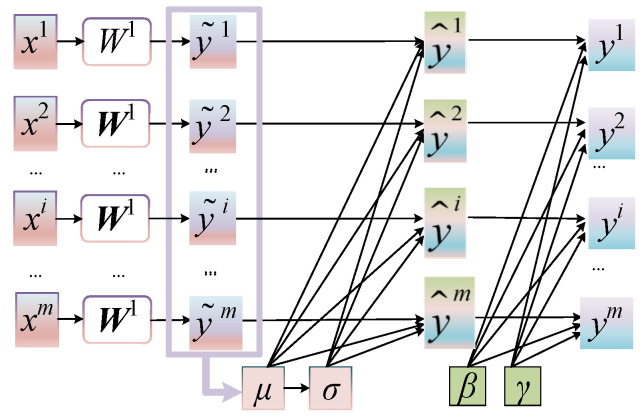
Operating principle of BN.

The covariate shift problem in statistical machine learning algorithms is universally known. To solve the problem, Sergey and Christian first proposed BN [30]. BN can reparametrize almost any network elegantly. First, it standardizes the output results of the hidden layer on batch, then scales and shifts it, and finally sends it to the next layer after being processed by the activation function, as shown in Figure 4.

For a layer with *m*-dimensional input *x* = (*x*^1^…*x^m^*), firstly, each scalar feature is normalized independently.

(6) $${\tilde{y}}^{i}=W^{1}x^{i},$$

(7) $${\hat{y}}^{i}=\frac{{\tilde{y}}^{i}-\mu ({\tilde{y}}^{i})}{\sqrt{\sigma {\left({\tilde{y}}^{i}\right)}^{2}+\varepsilon }},$$

(8) $$y^{i}=\gamma^{i}{\hat{y}}^{i}+\beta^{i},$$

where *μ* and *σ* are the mean and standard deviation, respectively. *ε* is the minimum quantity introduced to prevent division by zero. *γ* is the scale parameter, and *β* is the shift parameter.

Secondly, suppose that *x^i^* has *n* values and the minimum batch is {*x*_1…*n*_}, ${\hat{x}}_{1\ldots n}$ are the normalized values, and *y*_1… *n*_ denote the corresponding linear transformations. The BN transforms BN*_γ_*_, *β*_: *x*_1…*n*_→*y*_1... *n*_ can be displayed as follows:

(9) $$\mu (\{x_{1\cdots n}\})=\frac{1}{n}\sum_{k=1}^{n}x_{k},$$

(10) $$\sigma (\{x_{1\cdots n}\})=\frac{1}{n}\sum_{k=1}^{n}{\left(x_{k}-\mu (\{x_{1\cdots n}\})\right)}^{2},$$

(11) $${\hat{x}}_{k}=\frac{x_{k}-\mu (\{x_{1\cdots n}\})}{\sqrt{\sigma {\left(\{x_{1\cdots n}\}\right)}^{2}+\varepsilon }},$$

(12) $$y_{k}=\gamma \times {\hat{x}}_{k}+\beta .$$

Briefly speaking,

(13) $$y_{1\cdots n}={\mathrm{BN}}_{\gamma ,\beta }(x_{1\cdots n}).$$

Moreover, the backpropagation process of the gradient of loss *E* through BN transformation is as follows,

(14) $$\frac{\partial E}{\partial {\hat{x}}_{k}}=\frac{\partial E}{\partial y_{k}}\gamma ,$$

(15) $$\frac{\partial E}{\partial \sigma {\left(\{x_{1\cdots n}\}\right)}^{2}}=\sum_{k=1}^{n}\frac{\partial E}{\partial {\hat{x}}_{k}}(x_{k}-\mu (\{x_{1\cdots n}\}))\cdot [-\frac{1}{2}{\left(\sigma {\left(\{x_{1\cdots n}\}\right)}^{2}+\varepsilon \right)}^{\frac{3}{2}}],$$

(16) $$\frac{\partial E}{\partial \mu (\{x_{1\cdots n}\})}=\sum_{k=1}^{n}\frac{\partial E}{\partial {\hat{x}}_{k}}\frac{-1}{\sqrt{\sigma {\left(\{x_{1\cdots n}\}\right)}^{2}+\varepsilon }},$$

(17) $$\frac{\partial E}{\partial x_{k}}=\frac{\partial E}{\partial {\hat{x}}_{k}}\frac{-1}{\sqrt{\sigma {\left(\{x_{1\cdots n}\}\right)}^{2}+\varepsilon }}+\frac{\partial E}{\partial \sigma {\left(\{x_{1\cdots n}\}\right)}^{2}}\cdot \frac{2(x_{k}-\mu (\{x_{1\cdots n}\}))}{n}+\frac{\partial E}{\partial \mu (\{x_{1\cdots n}\})}\cdot \frac{1}{n},$$

(18) $$\frac{\partial E}{\partial \gamma }=\sum_{k=1}^{n}\frac{\partial E}{\partial y_{k}}\cdot {\hat{x}}_{k},$$

(19) $$\frac{\partial E}{\partial \beta }=\sum_{k=1}^{n}\frac{\partial E}{\partial y_{k}}.$$

The existence of the BN layer is equivalent to separating the mean and variance of the distribution from the weight. The distribution can be adjusted directly by adjusting the parameters of *γ* and *β*, which makes the coordination of distribution and weight easier. Fortunately, BN can avoid some of the drawbacks of SAE, so BNSAEs were ultimately chosen as the baseline methods for this article.

## 3. The Proposed Method

In the vibration-based bolt micro looseness monitoring method, firstly, the vibration exciter or hammer is used to excite the bolt joint structure, then the sensor is used to collect the vibration response signal, and finally, the signal analysis technology is used to analyze and extract some characteristic parameters as the bolt micro looseness monitoring index. The proposed method first establishes the characterization function to preliminarily extract the characteristics of the vibration response signal, and then the preliminarily extracted data is fed into the BNSAEs and identified according to the classification index of the corresponding looseness.

### 3.1. Characterization Function

Firstly, the power spectral density of the original response signal is estimated by pwelch [31], and the windowing function is the Hanning window. Then, the most important features of the response spectrum matrix are extracted by singular value decomposition (SVD). When the response spectrum matrix is directly decomposed by the SVD, the following expression is obtained.

(20) $$S_{yy}^{T}(f)=U\Lambda U^{H},$$

(21) $$\Lambda =\mathrm{diag}(\lambda_{1},\lambda_{2},\cdots ,\lambda_{N}),$$

where ***S****_yy_* is the response spectral density matrix, *f* is the frequency, and ‘T’ denotes the transposition of a matrix. ‘H’ denotes the conjugate transposition of the matrix, and ***U*** ∈ **C***^M×N^* denotes a unitary matrix that contains a singular value vector. *λ* is the eigenvalue, ***Λ*** ∈ **R***^N×N^* denotes the diagonal matrix that consists of real singular values.

According to the first-order eigenvalue of the singular values, define the characterization function.

(22) $$C\left(f\right)=\left\{\log[\lambda_{1}(f)]\right\}.$$

**Figure 5:**
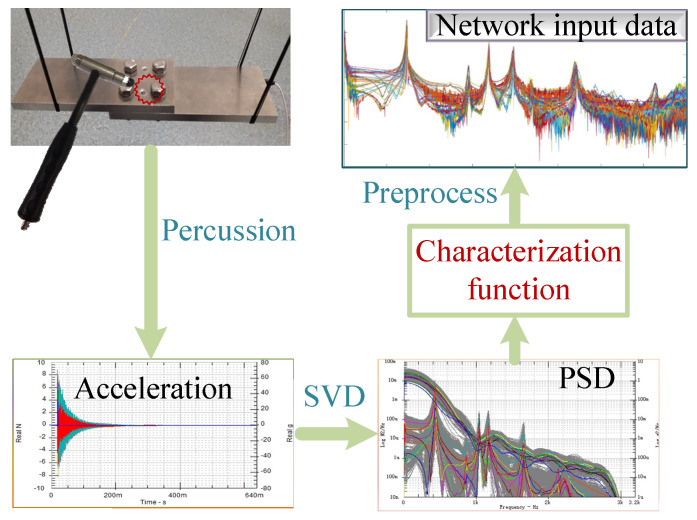
Flowchart of data preprocessing by characterization function.

Therefore, we use the characterization function to process the response signal to obtain the input dataset of the networks. See Figure 5.

### 3.2. Framework of BNSAEs

SAEs consist of several AEs and are highly efficient in feature extraction, dimension reduction, and classification projects [23,32]. Here, we apply the BN immediately before activating the layers of SAEs. According to Equation (13), **x** = ***C***(*f*), Equation (2) is replaced with,

(23) $$h_{i}^{1}=f_{h}({\mathrm{BN}}_{\gamma ,\beta }(W_{e}x_{i})),$$

(24) ReLU(x)=max(x,0),

The BN transform is used separately on each dimension of **W***_e_***x***_i_* with an independent pair of learned parameters *γ_i_* and *β_i_*. Where *f_h_* (▪) represents the ReLU function, which can perfectly alleviate the gradient vanishing problem. Herein, combine it with BN for a loose bolt system automatically. SAE progresses layer by layer until the end of the last AE training,

(25) $$h_{i}^{N}=f_{h}({\mathrm{BN}}_{\gamma ,\beta }(h_{i}^{N-1})).$$

**Figure 6:**
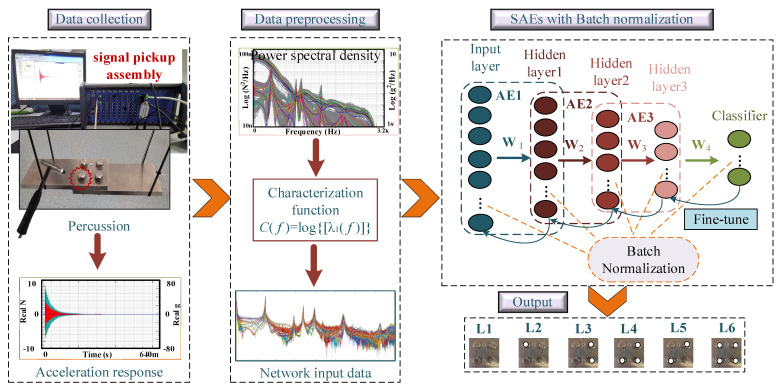
Flowchart of the proposed method.

Subsequently, the softmax classification layer containing the sample label is taken as the output layer of BN-DNNs, which is added after SAE, and then the BP algorithm is used to realize the reverse layer-by-layer parameter fine-tuning of BN-DNNs. Therefore, the BN-DNNs output calculated from the input signal **x** is:

(26) $$y=f(h^{N}),$$

(27) $$\mathrm{Softmax}(x_{i})=\frac{e^{x_{i}}}{\sum_{c=1}^{C}e^{x_{i}}},$$

where *f* (▪) represents the Softmax function [33], *x_i_* denotes the output of the *i*_th_ node, and *C* denotes the output nodes. The framework and illustration of the proposed method are depicted in Figure 6. The BNSAEs module is employed to extract invaluable features from each sample. The procedure can be represented as follows:

(1) The response signals of all bolt health conditions under different tightening torques were measured, and the signal according to *C*(*f*) to obtain the training set {x*^j^*,*l^j^*${\}}_{j=1}^{M}$ the training set {x}, and *M* means samples size, and x*^j^*∈R^n×1^ and *l^j^* are the *j*_th_ sample and the corresponding health label, respectively.
(2) Construction of DNNs with BNSAEs. Then, apply unmarked training set {x*^j^*${\}}_{j=1}^{M}$ as input to pre-training the BNSAEs layer-by-layer. Take the features learned from the previous layer as the input of the next layer, and repeat the above process till the training is completed.
(3) To minimize the error among the extracted features and looseness labels, the Back Propagation algorithm is employed to renew the weights and fine-tune the parameters of SAEs with a marked training set.
(4) The validity of the proposed method is proved by the testing set.

**Figure 7:**
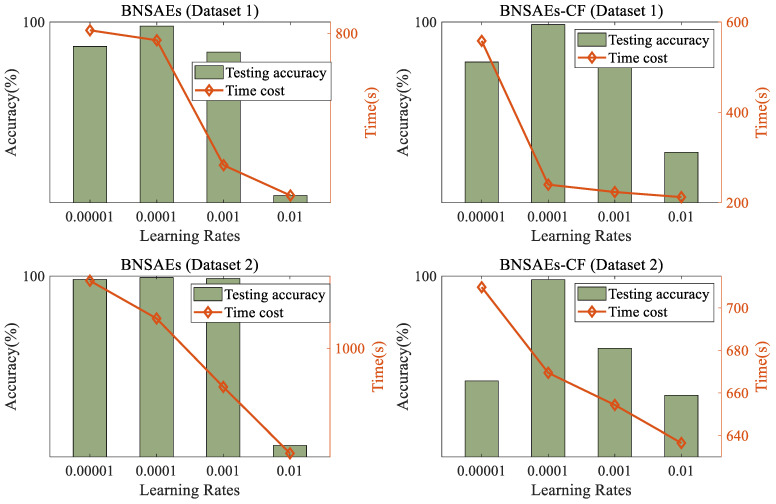
Comparison of Time Cost and Accuracy Based on BNSAEs with Different Learning Rates.

Perform 5 ablation experiments [12,13] were performed on the BNSAEs model using a typical bolted structure (Dataset 1), and the average time cost and accuracy of different learning rates are shown in Figure 7.

From the analysis of Figure 7, it can be seen that (1) when training based on the BNSAEs model with learning rates set to 1 × 10^−4^, both datasets have the highest testing accuracy, indicating that the model is optimal when the learning rate is set to 1 × 10^−4^. (2) As the learning rate of the model gradually increases, the time cost of the model gradually decreases, but the difference is not too large, indicating that the impact of changes in learning rate on time cost cannot be used as a key factor to judge the quality of the model. (3) Based on different training methods on two datasets, when the learning rate is the same, the testing accuracy of the BNSAEs-CF method is significantly higher than that of the BNSAEs method, indicating that the BNSAEs-CF method has higher recognition accuracy. (4) Based on different training methods on two datasets, when the learning rate is the same, the time cost of the BNSAEs-CF method is significantly lower than that of the BNSAEs method, indicating that the BNSAEs-CF method has lower computational cost and better real-time performance. Through ablation experiments, the structural parameters of the BNSAE model were finally determined, as shown in Table 1.

## 4. Experimental Verification

### 4.1. Dataset

**Figure 8:**
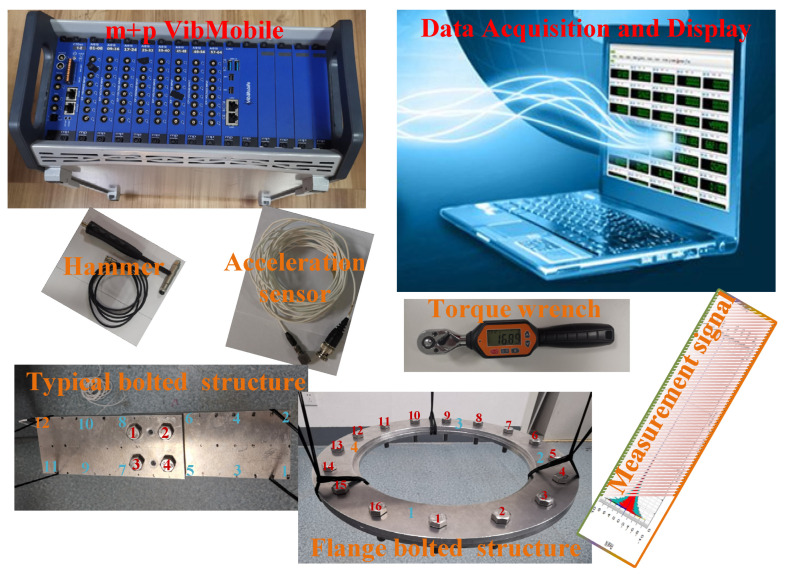
Experimental acquisition and measurement system.

The proposed method was assessed using the bolted connection model of the typical aircraft structure. Experimental acquisition and measurement system, as shown in Figure 8. The sensitivity of the impact hammer and the acceleration sensor are 2.54 mV/EU and 100.2 mV/EU, respectively. The sample rate is 6400 Hz, the useful bandwidth is 2500 Hz, the sampling point is 4096, the useful spectral line is 1600, and the sampling length is 0.63999 s. Numerals in different colors on the structure represent different meanings: red represents bolt numbers, blue represents hammering points, and yellow represents hammering points and response acquisition points. The material parameters of the structure are shown in Table 2.

First, the typical bolt structure is tested. We can tighten or loosen the bolts by using a torque wrench and set six different instances for each bolted connection under 4 different tightening torques, and there are 1200 samples. There are 200 samples for each looseness condition, and each sample consists of 4096 data points. For six different types of bolt health conditions: no looseness (NL), one bolt micro looseness (LB1), two bolts looseness in the vertical direction (LB2), three bolts looseness (LB3), two bolts looseness in the diagonal direction (LB4), and four bolts looseness (AL). Specifically, 0 represents no looseness, and 1 represents looseness. There are four forms of looseness of one bolt ([1 0 0 0 0] [0 1 0 0] [0 0 1 0] [0 0 0 1]), but the effect is the same, so the other three forms are represented by LB1, and the other conditions are the same. Similarly, we conducted experiments on the flange bolt structure, as shown in Table 3.

**Figure 9:**
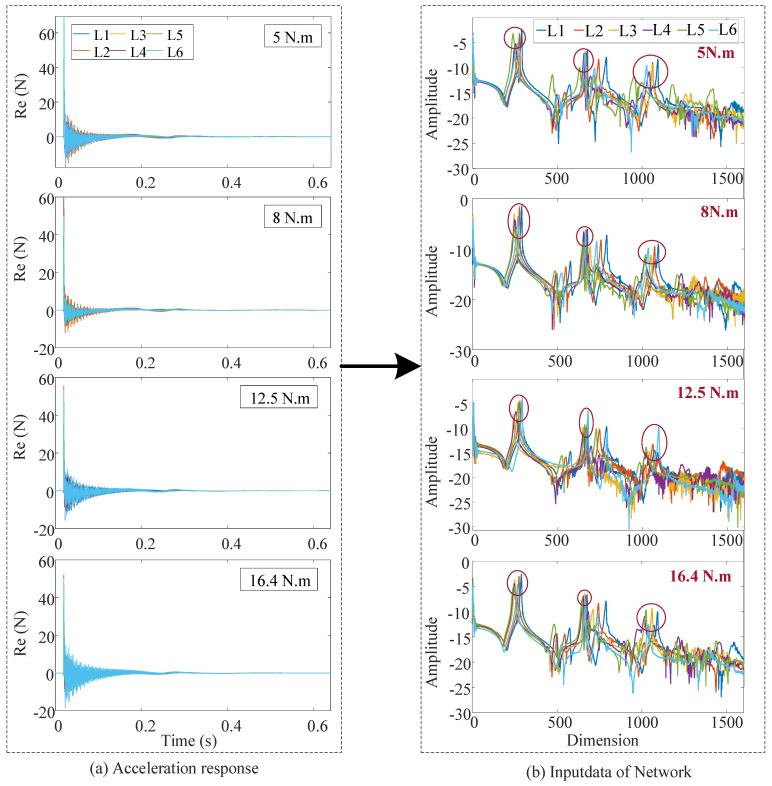
Signal processing of different health conditions under different tightening torques of Dataset 1.

According to the characterization function, the acceleration response signals (using a typical bolt structure dataset 1) under different tightening torques are preprocessed, and each sample contains 1600 data points. The comparison of data before and after preprocessing is shown in Figure 9. Analysis of Figure 9 reveals that: (1) Different types of bolt micro looseness under the same tightening torque are different; (2) the same type of bolt micro looseness under different tightening torques is also different; (3) After the characterization function processing, not only the required data characteristics are perfectly preserved, but also the data length accounts for only 0.39 of the time-domain signal length, which greatly saves the calculation and time cost; (4) In addition, after the processing of the characterization function, the characteristics of different bolt types are preliminarily extracted, which is equivalent to the preprocessing of data and is helpful for network training; (5) Overall, it can be seen that the effectiveness of the characterization function in data processing is intuitively demonstrated.

### 4.2. Model Training and Results

The BNSAEs possess five layers, and the count of neurons in the input layer is equivalent to the dimension of the input samples. Set the number of neurons in the hidden layers of the BNSAEs to 800, 400, and 100, respectively, according to the dimension reduction layer by layer, and the counts of output neurons are the same as the number of the health status of bolts. The activation function of the network is the ReLU function, the number of training iterations per layer is 100, the learning rate is 0.0001, the momentum is 0.05, and the batch size is 10.

Different methods are trained and compared to verify the advantages of the proposed method. Comparison with method 1: the network structure is SAEs without BN, and the input is the same as the proposed method, which is preprocessed by *C*(*f*). Comparison method 2: the network structure is the same as the proposed method, but the input is the original vibration response signal. Comparison method 3: the network structure is the same as that of Comparison method 1, and the input is the same as that of comparison method 2. The results are shown in Table 4.

(28) $$T_{1}<1.5\times T_{2}.$$

When the time cost is acceptable, or the difference is small, the completion quality of the project will be used as the evaluation standard in actual engineering, which is a convention. Therefore, this article assumes that accuracy is the only evaluation criterion when the time cost satisfies Equation (28).

**Figure 10:**
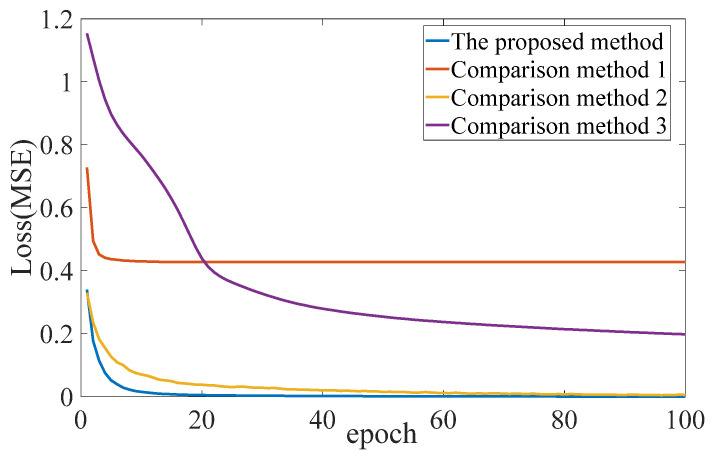
Training error of different methods of Dataset 1.

Now, we use Dataset 1 for specific analysis. The loss curves of different methods based on Dataset 1 are shown in Figure 10. Subsequently, an analysis of Table 4 and Figure 10 reveals that (1) when the original vibration signal is used as the input dataset, the recognition accuracy of Comparative Method 2 is over 99%, which is much higher than that of Comparative Method 3, with an accuracy of less than 50%. In addition, the time cost of comparative method 2 is lower than that of comparative method 3. The above demonstrates the effectiveness of the BNSAEs model. (2) When the preprocessed dataset is used as input, the proposed method achieves a recognition accuracy of 100%, which is higher than the comparison method 1, indicating the effectiveness of the BNSAEs model. (3) When the network models are all BNSAEs, the proposed method for preprocessing the dataset has a recognition accuracy of 100%, which is higher than the comparison method 2 (with a recognition accuracy of 99–99.7%), and the time cost of the proposed method is about 0.3 times that of the comparison method 2, indicating the effectiveness of feature function preprocessing of data. (4) The MSE of the proposed method iteration in step 100 is 0.0002297, which is much smaller than that of Comparison method 1 in step 100 iteration, which is 0.4276, indicating that the proposed method has a fast convergence speed. (5) The accuracy of the proposed method is significantly better than that of other methods. (6) Except that the time cost of the proposed method is 239.9550 s, which is slightly higher than that of Comparison method 1, other aspects show that the proposed methods are optimal. The addition of BN will inevitably increase the time cost, but it is acceptable. (7) When the time cost is acceptable, or the difference is small, the accuracy rate is used as the standard to evaluate various methods. (8) As explained above, BN makes the convergence speed of the network faster and solves the problems of gradient disappearance and over-fitting. (9) The loss curve of the proposed method is smoother than that of Comparison method 2, and the time cost of the proposed method is also significantly lower than that of Comparison method 2. Herein, using *C*(*f*) to preprocess the original response signal not only simplifies the data structure but also ensures the integrity of features, which is conducive to network training and reduces the time cost. (10) In summary, the proposed method is the best, followed by Comparison method 2, then Comparison method 1, and Comparison method 3 is the worst.

**Figure 11:**
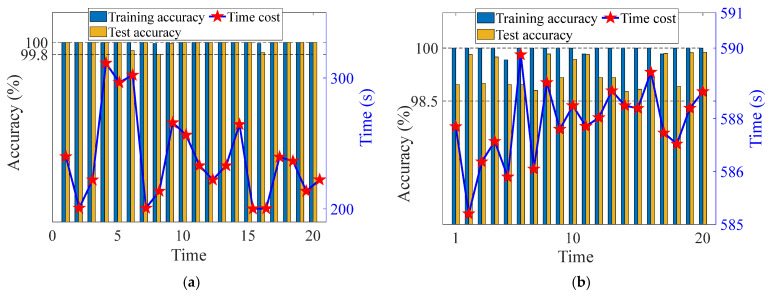
Accuracy of different methods based on Dataset 1. (**a**) The proposed method (**b**) Comparison method 2.

**Figure 12:**
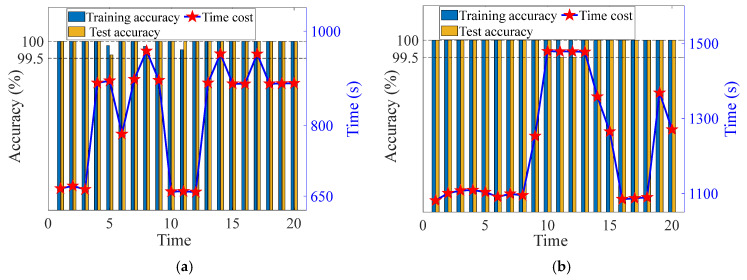
Accuracy of different methods based on Dataset 2. (**a**) The proposed method (**b**) Comparison method 2.

**Figure 13:**
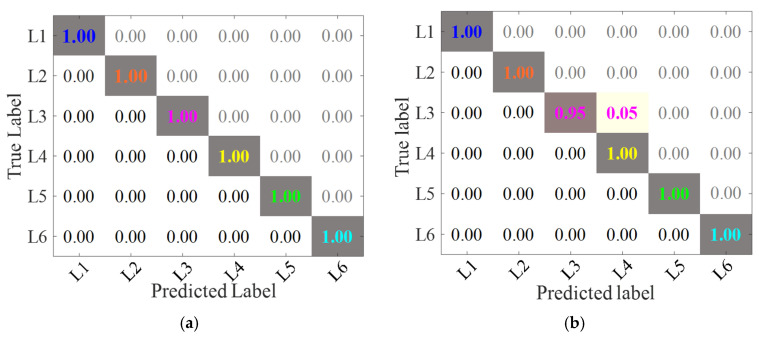
Confusion matrix of different methods based on Dataset 1. (**a**) The proposed method (**b**) Comparison method 2.

**Figure 14:**
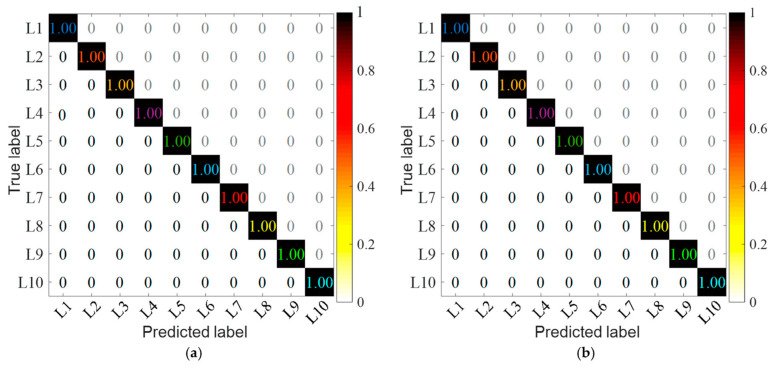
Confusion matrix of different methods based on Dataset 2. (**a**) The proposed method (**b**) Comparison method 2.

Additionally, to eliminate the influence of randomness, each group of trials was conducted 1000 times, respectively. In order to further analyze the accuracy of the proposed method and Comparison method 2, the accuracy of training and testing was randomly selected 20 times, as shown in Figure 11 and Figure 12. The confusion matrix of the proposed method and comparison method 2 are shown in Figure 13 and Figure 14. Analysis of Figure 11, Figure 12, Figure 13 and Figure 14 reveals that: (1) The average training accuracy of the proposed method is 99.98%, the average test accuracy is 99.77%, and all of them are greater than 99.8%. (2) The average training accuracy of Comparison method 2 is 83.69%, and the average test accuracy is 82.56%. (3) It is obvious that the proposed method identifies confusion matrices for different datasets where all diagonals are 1. However, in Comparison method 2, in the confusion matrices for different datasets, only L3 in Figure 13b is 0.95, and not all diagonals are 1. (4) In summary, the proposed method is optimal.

## 5. Conclusions

An automated bolt micro looseness monitoring method using BNSAEs is proposed and applied to the health monitoring of the two most commonly used bolt structures in aircraft engines. Among them, the two structures are typical bolted structures and flange bolted structures. The proposed method first preprocesses the response signal and then realizes the classification and recognition of bolt micro looseness through the BNSAEs network. Experimental results show that the proposed method has the strengths of fast convergence and high recognition accuracy. Particularly, it prepares an innovative method for the application of bolt micro looseness monitoring in engineering.

(1) The proposed method has a smoother training loss curve compared to the comparative method 2. The essential difference between the two methods lies in the difference in the input dataset. The input dataset of the proposed method is preprocessed with characterization functions (C (f)) to give the network structure fewer layers and feature parameters, thereby simplifying the model and reducing the time cost, resulting in stronger real-time performance. The input dataset for comparison method 2 is the original vibration signal, with a high signal-to-noise ratio and long training time.

(2) To eliminate the randomness, each group of trials with different methods was conducted 1000 times, respectively. The average test accuracy of the proposed method is 99.77%, which is significantly better than that of Comparison method 2 (the average test accuracy is 82.56%). Even if the time cost of the proposed method is slightly higher than that of comparison method 2, the proposed method has higher accuracy. It provides an elegant manner to achieve fast training of the deep architecture and realize high-efficiency bolt micro looseness detection.

(3) Compared with traditional bolt loosening detection methods, the proposed method directly identifies the vibration response signal without complex calculation and excessive consideration of the deployment of measuring points, which has a low economy and labor cost and can realize automatic monitoring. And with the addition of intelligent detection equipment, automatic monitoring can also be achieved.

## Figures and Tables

**Table 1:** The structural parameters of BNSAEs.

Layer	Parameters	Activation Function	Learn Rate	Optimization
AE1	(1600, 800)	ReLU	1 × 10^−4^	BN
AE2	(800, 400)	ReLU	1 × 10^−4^	BN
AE3	(400, 100)	ReLU	1 × 10^−4^	BN
Classification layer	(100, output)	Softmax	1 × 10^−4^	-

**Table 2:** Material parameters of the plate structure.

Elastic Modulus *E* (GPa)	Poisson’s Ratio *μ*	Density *ρ* (kg/m^3^)	Hardness *H* (MPa)	Roughness *R_a_* (μm)
68.9	0.33	2718.3	95	3

**Table 3:** Description of bolt micro looseness datasets.

Dataset	Sample Number	Loose Form	Label
Typical bolted structure (Dataset 1)	200	No loose bolts ([0 0 0 0])	L1
	200	1 loose bolts ([1 0 0 0])	L2
	200	2 loose bolts_ vertical direction ([1 1 0 0])	L3
	200	3 loose bolts ([1 1 1 0])	L4
	200	2 loose bolts- diagonal direction ([1 0 1 0])	L5
	200	4 loose bolts ([1 1 1 1])	L6
Flange bolted structure (Dataset 2)	100	All bolts are tightened ([0 0 0 0 0 0 0 0 0 0 0 0 0 0 0 0])	L1
	100	No.1 bolt is loose and other bolts are tightened ([1 0 0 0 0 0 0 0 0 0 0 0 0 0 0 0])	L2
	100	No.1 and No.9 bolts are loose and other bolts are tightened ([1 0 0 0 0 0 0 0 1 0 0 0 0 0 0 0])	L3
	100	No.1, No.5 and No.9 bolts are loose and other bolts are tightened ([1 0 0 0 1 0 0 0 1 0 0 0 0 0 0 0])	L4
	100	No.1, No.5, No.9 and No. 13 bolts are loose and other bolts are tightened ([1 0 0 0 1 0 0 0 1 0 0 0 1 0 0 0])	L5
	100	No.1, No.3, No.5, No.9 and No. 13 bolts are loose and other bolts are tightened ([1 0 1 0 1 0 0 0 1 0 0 0 1 0 0 0])	L6
	100	No.1, No.3, No.5, No.9, No.11 and No. 13 bolts are loose and other bolts are tightened ([1 0 1 0 1 0 0 0 1 0 1 0 1 0 0 0])	L7
	100	No.1, No.3, No.5, No.7, No.9, No.11 and No. 13 bolts are loose and other bolts are tightened ([1 0 1 0 1 0 1 0 1 0 1 0 1 0 0 0])	L8
	100	No.1, No.3, No.5, No.7, No.9, No.11, No. 13 and No. 15 bolts are loose and other bolts are tightened ([1 0 1 0 1 0 1 0 1 0 1 0 1 0 1 0])	L9
	100	All bolts are loose ([1 1 1 1 1 1 1 1 1 1 1 1 1 1 1 1])	L10

**Table 4:** Comparison of results from identifying different datasets using different methods.

Dataset	Methods	The Proposed Method	Comparison Method 1	Comparison Method 2	Comparison Method 3
Dataset 1	Time cost (s)	239.9550	200.05	793.8670	270.0420
	Training accuracy (%)	100	83.33	99.52	25.5
	Test accuracy (%)	100	83.33	99.17	46
Dataset 2	Time cost (s)	669.4670	176.83	1024.8	524.05100
	Training accuracy (%)	100	82	99.69	40
	Test accuracy (%)	100	82	99.48	40

## Data Availability

The data presented in this study are available on request from the corresponding author.
